# Complete Genome Sequence of the Industrial Strain *Gluconobacter oxydans* H24

**DOI:** 10.1128/genomeA.00003-13

**Published:** 2013-02-21

**Authors:** Xin Ge, Yan Zhao, Wei Hou, Weicai Zhang, Weiwei Chen, Jianhua Wang, Nan Zhao, Jian Lin, Wenxi Wang, Mengxia Chen, Qingge Wang, Yinghui Jiao, Zhigang Yuan, Xianghua Xiong

**Affiliations:** a Laboratory of Microorganism Engineering, Beijing Institute of Biotechnology, Beijing, China; b Central Laboratory, First Affiliated Hospital of Xiamen University, Xiamen, China; c School of Preclinical Medicine, Guangxi Medical University, Nanning, China; d School of Life Science, Biochemical Pharmaceutical, Shenyang Pharmaceutical University, Shenyang, China; e Weisheng Pharmaceutical Co. Ltd. and Shijiazhuang Pharma Group, Shijiazhuang, China

## Abstract

*Gluconobacter oxydans* is characterized by its ability to incompletely oxidize carbohydrates and alcohols. The high yields of its oxidation products and complete secretion into the medium make it important for industrial use. We report the finished genome sequence of *Gluconobacter oxydans* H24, an industrial strain with high l-sorbose productivity.

## GENOME ANNOUNCEMENT

*Gluconobacter oxydans* is a Gram-negative bacterium belonging to the family *Acetobacteraceae*. This bacterium has a number of membrane-bound dehydrogenases involved in many oxidation reactions, which bring about the incomplete oxidation of sugars, alcohols, and acids. Owing to its incomplete oxidation and almost complete secretion, *G. oxydans* is widely used in industrial production (1). *Gluconobacter oxydans* H24, used in the industrial production of vitamin C in China, is responsible for the oxidization of d-sorbitol into l-sorbose. It has been consecutively optimized for several decades by mutation from a wild-type strain to improve its production of l-sorbose and its tolerance to substrate and product. Here, the entire genome of *G. oxydans* H24 was sequenced to elucidate the details of the metabolic pathway of d-sorbitol.

The complete genome sequence of *G. oxydans* H24 was determined at the Beijing Genome Institute (BGI) (Shenzhen, China) using Solexa technology. Draft assemblies were based on 925Mb total reads. All reads provided 242-fold coverage of the genome. The initial assembly of Solexa sequencing data into 24 contigs was provided by BGI, whereas the assembly of contigs into 15 scaffolds was performed using Short Oligonucleotide Alignment Program (SOAP) *denovo* software (http://soap.genomics.org.cn) (2). Physical gaps, repeats, and assembly ambiguities were closed and corrected by custom primer walks or by long-distance PCR amplification and Sanger sequencing. Protein-coding genes were predicted using Glimmer 3.0 (3). rRNAs and tRNAs were predicted using rRNAmmer (4) and tRNAscan-SE (5), respectively. Genes were annotated through BLASTp searches against the nonredundant (NR), Swiss-Prot, TrEMBL, Kyoto Encyclopedia of Genes and Genomes (KEGG), Clusters of Orthologous Groups (COG), and Gene Ontology (GO) databases (6–8).

The complete genome of *G. oxydans* H24 consists of a circular chromosome and a plasmid. The chromosome is composed of 3,602,424 bp, with a G+C content of 56.25%. The plasmid contains 213,808 bp, with a G+C content of 56.14%. There are a total of 3,732 putative open reading frames (3,469 in the chromosome and 263 in the plasmid), yielding a coding intensity of 89.86%. A total of 59 tRNA-encoding genes and 5 16S-23S-5S rRNA-encoding operons were identified. The genome sequences of *Gluconobacter oxydans* 621H (GenBank accession no. CP000004 to CP000009) were used as the reference (9).

The most significant feature of *G. oxydans* H24 is its high l-sorbose productivity. Two different membrane-bound, and one cytoplasmic, sorbitol dehydrogenases were identified from genome information: pyrroloquinoline quinone-dependent d-sorbitol dehydrogenase (PQQ-SLDH) (*sldhAB*) (10), flavin adenine dinucleotide-dependent d-sorbitol dehydrogenase (FAD-SLDH) (*sldhSLC*) (11), and NADP-dependent d-sorbitol dehydrogenase (NADP-SLDH) (*sldH*) (12). A comparison of three sorbitol dehydrogenase sequences with those of *G. oxydans* 621H indicated that the homology was 79.4%, 63.6%, and 29.2%, respectively. The gene cluster responsible for the synthesis of the cofactor PQQ (*pqqABCDE*, 3,137 bp) was also found (13). In addition, several genes encoding sorbose dehydrogenase (14), sorbose reductase (15), sorbosone dehydrogenase (14), glucose dehydrogenase, and other enzymes were annotated.

In summary, the genome sequence of *G. oxydans* H24 and its curated annotation are important assets for understanding better the physiology and metabolic potential of *G. oxydans*, and they will open up new opportunities to understand the functional genomics of this species.

### Nucleotide sequence accession numbers.

The nucleotide sequence was deposited in the GenBank database under the accession no. CP003926 and CP003927. The version described in this paper is the first version.
